# Horse Teeth

**Published:** 1881-02

**Authors:** 


*Horse Teeth.* A treatise on their mode of development, by William H. Clark. Published by the Author, New York, 1881.

This work treats of the physiological relations, anatomy, microscopical character, pathology and veterinary operations pertaining to such structures, based on the works of well known odontologists and veterinary surgeons. A vocabulary of the medical and technical words in use is added.

